# Holographic Spectroscopy: Wavelength-Dependent Analysis of Photosensitive Materials by Means of Holographic Techniques

**DOI:** 10.3390/ma6010334

**Published:** 2013-01-23

**Authors:** Kay-Michael Voit, Mirco Imlau

**Affiliations:** School of Physics, Osnabrück University, Barbarastr. 7, Osnabrück 49069, Germany; E-Mail: mimlau@uos.de

**Keywords:** holographic spectroscopy, holographic materials, coupled-wave theory, mixed gratings, out-of-phase gratings, Borrmann effect, beam-coupling analysis, diffraction efficiency, **PACS** 42.40.-i, 42.40.Lx, 42.40.Pa, 42.40.My, 42.65.-k, 42.70.Ln

## Abstract

Holographic spectroscopy is highlighted as a powerful tool for the analysis of photosensitive materials with pronounced alterations of the complex permittivity over a broad range in the visible spectrum, due to the advances made both in the fields of advanced holographic media and highly tunable lasers systems. To analytically discuss consequences for in- and off-Bragg reconstruction, we revised Kogelnik’s coupled wave theory strictly on the basis of complex permittivities. We extended it to comply with modern experimental parameters such as out-of-phase mixed holograms and highly modulated gratings. A spatially modulated, wavelength-dependent permittivity that superimposes a spatially homogeneous wavelength-dependent ground state spectrum is taken into account for signal wave reconstruction with bulky elementary mixed gratings as an example. The dispersion characteristics of the respective diffraction efficiency is modelled for color-center-absorption and absorption of strongly localized carriers. As an example for the theoretical possibilities of our newly derived set of equations, we present a quantitative analysis of the Borrmann effect connected to out-of-phase gratings, providing easier and more intuitive methods for the derivation of their grating parameters.

## 1. Introduction

Holography [1,2] provides impressive tools for the analysis of photosensitive materials at optical frequencies: test holograms can be recorded by optical means, and phase-front as well as amplitude-distribution of the reconstructed signal waves are detected.

Applying an appropriate theoretical model for the analysis yields the modulation depth Δϵ of the material’s complex permittivity modulation and, if present, of the phase-shift between refractive index and absorption grating Φ. Thereby, the photo-physical processes underlying the holographic recording of optically induced gratings, *i.e*., the materials’ photosensitivity in general, can be studied. (Such processes can be rather complex, such as the photorefractive effect that combines optical excitation of charge carriers in wide-band gap dielectrics, charge transport phenomena, the build-up of electric space-charge fields due to re-trapping in dark regions of the fringe pattern and the modulation of the refraction index via the Pockels effect.) It is the starting point for a targeted design of hologram recording media. Also, the optimum boundary conditions for hologram recording, taking into account the large variety of upcoming applications of modern holography in photonics [3,4], such as for instance real-time holographic displays, can be determined.

Prominent examples for the successful application of holography for material analysis are photorefractive crystals, *i.e*., crystals that obey the photorefractive effect for hologram recording. A second example is molecular holographic media built from transition-metal-compounds. Here, the targeted design of hologram recording materials related to a broad range of parameters including photosensitivity, spectral sensitivity and hologram lifetime has been demonstrated [5,6].

The correct analysis of phase and amplitude of the reconstructed signal wave from the test hologram is the key point of material characterization by means of holography. Boundary conditions for the derivation of the analysis have to be chosen adequately to experimental conditions and material parameters. Several publications in literature face this type of analysis in detail for isotropic media [7,8,9,10,11,12,13,14,15,16] with Kogelnik’s *coupled wave theory for thick hologram gratings* [17] being the most recognized one. Anisotropic media were studied thoroughly by Montemezzani and Zgonik [18] and Sturman *et al*. [19] by vectorial theories and consideration of the tensorial properties of the permittivity. This enabled the analysis of such important classes of photosensitive materials as semiconductors and sillenites. In other words, the underlying theoretical problem has been solved decades ago and the derived set of formula is applied to a manifold of hologram recording media and phenomena.

However, some of the chosen boundary conditions do not fully comply with experimental parameters of modern holographic recording setups or the features of advanced hologram recording media. From the experimental point of view, tunable lasers have become state-of-the-art, thus, admitting hologram reconstruction over a broad range in the optical spectrum and the application of the holographic principle for spectroscopy. Advanced hologram recording media, such as garnets, nematic liquid crystal cells, photopolymers or molecular crystals, feature pronounced alterations of the complex dielectric permittivity. Some of these materials also show an arbitrary phase shift between the modulation of the real and the imaginary part of the permittivity. These conditions have been described by Guibelalde [7], but the derived equations show a high grade of formal complexity that prevents most further analysis [20].

For a broader range of applications, a more manageable set of formula is preferable, that particularly allows for a higher flexibility with respect to the set of boundary conditions. We have revised the derivation of the coupled wave theory accordingly by keeping the permittivity and its optically induced alteration as complex measure throughout the manuscript. We use the scalar permittivity. Thus, the theory presented is applicable to isotropic media and materials and setups in which an effective scalar permittivity may be assumed and the modulated permittivity tensor maintains diagonal form [18].

It turns out that the derived set of formula enables the analytical expression of measures that have not been reported in literature, so far—while classical measures still are involved. As an example, we introduce an analytically simple expression for the intensity ratio of the plus- and minus-first-order diffracted waves (*Borrmann*-effect), which allows easier characterisation of complex mixed grating structures. Moreover, a quantitative analysis for hologram reconstruction taking into account angular detuning and mixed, out-of-phase gratings is presented (*rocking curve*). All measures are presented as wavelength-dependent properties, thus enabling the analysis of the dispersive features of hologram reconstruction in presence of a pronounced spectrum of the ground state absorption. While experimental basics have been described in [21], a complete theoretical analysis of this holographic spectroscopy method is not yet present in literature. The dispersion related features are highlighted with hologram reconstruction at test holograms recorded via dopants and via small bound polarons as an example.

## 2. Recording a Test Hologram and the Properties of Its Permittivity

### 2.1. Recording of a Test Hologram

The superposition of two identical, mutually tilted coherent plane (or spherical) waves is a simple way to record test holograms. The respective fringe pattern is a one-dimensional (or radially symmetrical) intensity modulation aligned parallel to the samples’ entrance surface. Maxima and minima refer to constructive and destructive interference. From the point of view of the holographic principle, the two indistinguishable waves can be assigned unambiguously to reference (R-) and signal (S-) waves by choosing one of the beams for signal wave reconstruction. The signal is regarded as a point source positioned at infinite distance from the hologram recording medium.

**Figure 1:**
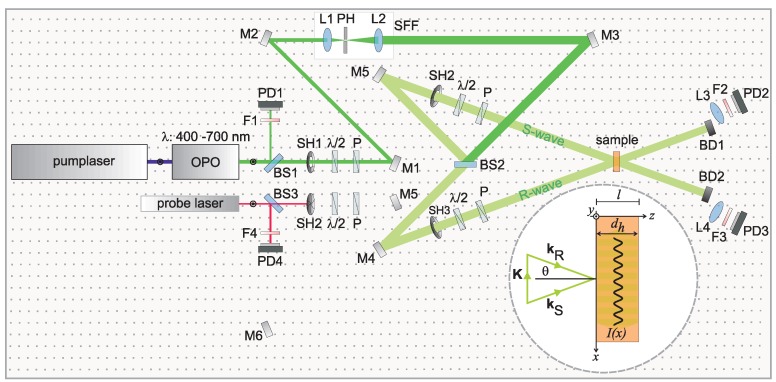
Sketch of an optical setup for the recording of a test hologram: reference (R) and signal (S) waves that are mutually coherent and feature a flat-top intensity distribution and planar wavefronts, superimpose in a photosensitive medium of thickness *l* and generate a sinusoidal intensity distribution. M1–M6: mirrors; L1–L4: lenses; BS1–BS3: beam splitters, F1–F4: filters, P: polarizers; λ/2: half-wave retarder plates; SH1–SH3: computer-controlled beam shutters; PD1-PD4: Si-PIN diodes; BD1,BD2: beam dumps; SFF: spatial frequency filter, PH: pinhole. The direction of the corresponding wavevectors of the recording beams ($k_{R}$, $k_{S}$) and the intensity distribution with respect to cartesian coordinates I(x) is depicted in the inset.

The corresponding optical setup for the case of plane phase fronts is depicted in Figure 1. Monochromatic laser light of a coherent, tunable laser source with flat-top intensity profile and planar wave-front is split into two identical beams (In case that a Gaussian intensity profile is present at the laser source output, an optical system for the transfer of Gaussian to flat-top-intensity profile [22] can be included in the setup). The symmetric configuration of the beam paths secures equal optical path lengths for R- and S-waves measured between the beamsplitter BS2 and the hologram recording medium (sample). At least, a path length match within the coherence length of the laser is required. Together with an adjustment of the beam intensities such that $I_{R}=|R_{0}{|}^{2}=I_{S}={\left|S_{0}\right|}^{2}$ as well as a precise parallel adjustment of the electric field vectors $e_{R}∥e_{S}$ via the polarizers P, a fringe pattern with maximum modulation depth $m=2e_{R}e_{S}|R_{0}S_{0}\left|/(\right|R_{0}{|}^{2}+S_{0}{|}^{2})=1$ is obtained. Here, $R_{0},S_{0}$ denote the electric field amplitudes of the R- and S-waves outside the medium. Recording of the hologram can be controlled via the shutter SH1.

We note that the homogeneity of the intensity profiles of the recording beams is optimized via the spatial frequency filter SFF (lenses L1 and L2, and pinhole PH) that represents a low-pass optical filter by positioning a pinhole (≪100 *μ*m) precisely within the Fourier plane of lens L1 (For the case of intense ns-pulses, the pinhole needs to be placed within an evacuated chamber in order to suppress plasma formation.) The precise adjustment of lens L2 is mandatory to keep the planar wavefront (This can be controlled simply by optical inspection of an interference pattern generated by a plane parallel plate inserted into the beam path and slightly tilted with respect to the beam direction. Alternatively, a Shack Hartmann wavefront sensor may be used).

The spatial intensity modulation that results from the interference between the R- and S-waves is aligned within the plane of incidence and orthogonal to the samples’ normal, *i.e*., along *x*-direction according to the inset of Figure 1. It represents a one-dimensional sinusoidal intensity distribution best described by $I\left(x\right)=|R_{0}+S_{0}{|}^{2}=\left(\right|R_{0}{|}^{2}+|S_{0}{|}^{2})\left[1+m\cos\left(Kx+\Phi_{0}\right)\right]$ with the modulation depth 0≤m≤1, the spatial frequency of the grating K=|K|=2π/Λ, and the wavelength related to the intensity modulation Λ ($K=k_{R}-k_{S}$, see inset of Figure 1). Here, $k_{R}$ and $k_{S}$ denote the wavevectors of the R- and S-wave with $|k_{R}\left|=\right|k_{S}|=2\pi /\lambda$. The wavelength Λ=λ/2sin(θ) is determined by the recording wavelengths and the angle of incidence $\theta =\theta_{R}=\theta_{S}$ that is measured with respect to the samples’ normal. The phase position $\Phi_{0}$ takes into account the relative phase adjustment of the R- and S-waves. Here, it is independent on time ($\Phi_{o}\left(t\right)=\Phi_{0}$) because of $\lambda =\lambda_{R}=\lambda_{S}$.

The characteristics of this optical setup comply with the simplifications of the theoretical analysis shown in Table 1.

### 2.2. Properties of the Permittivity of the Test Hologram

The analysis of the interaction of a coherent light wave with the test hologram starts with the Maxwell equations, particularly, by deducing the Helmholtz equation

(1) $$\Delta E\left(r\right)+k_{0}^{2}\hat{\epsilon }\left(r,\lambda \right)\mu E\left(r\right)=0$$

where E(r) is the electric field component perpendicular to the gradient of ϵ(r,λ), (The given equation holds true for most experimental setups, where ϵ(r,λ) is modulated parallel to the surface of the optical table and E is perpendicular to the table. In any other case, additional terms may have to be considered in the wave equation due to the anisotropic medium.) $k_{0}$ is the wavenumber in vacuum and ϵ(r,λ) is the spatially dependent absolute permittivity. The relative magnetic permeability $\mu_{r}$ is assumed to be unity. Common experimental media, however, are described in terms of the (real) refractive index and the absorption. All these quantities may strongly depend on the wavelength of the interacting electromagnetic wave and thus may be examined using spectroscopic methods.

In contrast to former derivations, we assumed the refractive index and the absorption index to be the fundamentally modulated quantities. This is justified by the fact that the relative complex permittivity ϵ(λ) is the natural quantity of Equation (1).

**Table 1:** Characteristics of the optical setup and their relation to simplifications made in the theoretical analysis.

Characteristic of the optical setup	Simplification for the theoretical analysis
hologram recording is performed with equal wavelengths of R- and S-waves *degenerate wave-mixing*)	$\lambda_{R}=\lambda_{S}$, *i.e*., $|k_{R}\left|=\right|k_{S}|=2\pi /\lambda$
hologram recording is performed by the superposition of planar wave fronts	sinusoidal permittivity modulation *
R- and S-waves feature flat-top intensity profile	the electric field amplitudes are constant within the beam paths and the beam is assumed to have infinite diameter
hologram recording is performed with equal directions of the electric field vectors of R- and S-wave and with equal intensities	$|R_{0}{|}^{2}={\left|S_{0}\right|}^{2}$, *i.e*., the modulation depth becomes unity (m=1), $e_{R}\cdot e_{S}=1$
hologram recording is homogeneously over the entire volume, *i.e*., exponential decrease of the grating parameters in *z*-direction is excluded (cf. [9]) **	permittivity modulation is not *z*-dependent
wave vector of the hologram is directed perpendicularly to the samples’ normal	permittivity modulation is aligned parallel to *x*-axis (*unslanted* hologram)

* Under the given conditions, a sinusoidal modulation is realized if hologram recording responses linear to the intensity of the recording beam superposition of planar wave fronts. However, at large modulation depth, the recording of holographic gratings is typically nonlinear [23,24]. In these cases, the permittivity modulation will not be sinusoidal, but a sine of the corresponding periodicity will usually be the dominating Fourier component. In the following sections, we will argue that within limits, the derived theory is still applicable then; ** Simply assuming a smaller effective grating depth than the crystal has shown the same effects in praxis.

Let us first have a look at the connection to the refractive index *n* and the absorption index *κ* with regard to a sinusoidally modulated mixed grating with a common grating vector K, but potentially different phase shifts $\Phi_{n}$ and $\Phi_{\kappa }$ (out-of-phase gratings) (The phase shifts are given in relation to the origin that is defined by the interference pattern of two incident beams without phase shifts):

(2) $$\begin{array}{c} n\left(r,\lambda \right)=n_{0}\left(\lambda \right)+n_{1}\left(\lambda \right)\cos\left(K\cdot r+\Phi_{n}\right) \end{array}$$

(3) $$\begin{array}{c} \kappa \left(r,\lambda \right)=\kappa_{0}\left(\lambda \right)+\kappa_{1}\left(\lambda \right)\cos\left(K\cdot r+\Phi_{\kappa }\right) \end{array}$$

where $K={\left(K_{x},0,0\right)}^{t}$ is the grating vector of a non-slanted grating. As n(r,λ)=n(r,λ)(1+iκ(r,λ)) is the complex refractive index and $\epsilon \left(r,\lambda \right)=n{\left(r,\lambda \right)}^{2}$ (assuming the permeability $\mu_{r}=1$ and frequencies in the optical range), this results in

(4) $$\begin{array}{cc} \epsilon (r,\lambda ) & ={n_{0}\left(\lambda \right)}^{2}+2n_{0}\left(\lambda \right)n_{1}\left(\lambda \right)\cos\left(K\cdot r+\Phi_{n}\right)\\ & -2{n_{0}\left(\lambda \right)}^{2}\kappa_{0}\left(\lambda \right)\kappa_{1}\left(\lambda \right)\cos\left(K\cdot r+\Phi_{\kappa }\right)\\ & +i[2{n_{0}\left(\lambda \right)}^{2}\kappa_{0}\left(\lambda \right)+2{n_{0}\left(\lambda \right)}^{2}\kappa_{1}\left(\lambda \right)\cos\left(K\cdot r+\Phi_{\kappa }\right)\\ & +4n_{0}\left(\lambda \right)n_{1}\left(\lambda \right)\kappa_{0}\left(\lambda \right)\cos\left(K\cdot r+\Phi_{n}\right)] \end{array}$$

We have omitted several terms in Equation (4) for two reasons: 1. The first class are terms that are nonlinear in $\kappa_{0}$. These terms are left out as $\kappa_{0}\ll 1$. (In the optical range, if $\kappa_{0}\approx$1, $\alpha_{0}\approx 10^{8}\;{\text{m}}^{-1}$, according to $\alpha =2k_{0}n_{0}\kappa_{0}$. Compared with common experimental values in the order of magnitude of $10^{2}$, this can be considered extremely high, thus justifying the assumption of $\kappa_{0}\ll 1$.) We like to note, however, that they can be incorporated into further calculation at the cost of slightly higher complexity but without generating new problems, as they are still proportional to $\cos(K\cdot r+\Phi_{\kappa })$ or $\cos(K\cdot r+\Phi_{n})$. 2. The second class consists of terms nonlinear in $\kappa_{1}$, $n_{1}$ and thus in a mixture of these cosine terms. Here, we can argue again that $\kappa_{1}$, $n_{1}\ll 1$, *i.e*., the light induced alterations are small, but a more convenient argument will be presented in the later discussion in Section 3.2.

The first cosine term in Equation (4) of the real part is the refractive index grating depending on $n_{1}$ and the first one in the imaginary part is the absorption grating depending on $\kappa_{1}$. Additionally, there is a real term which acts like a refractive-index grating though it is governed by $\kappa_{1}$, and carries the phase shift of the absorption grating. Furthermore, there is an imaginary one which correspondingly acts like an absorption grating but is proportional to $n_{1}$ and carries the phase shift of the refractive index grating. However, these terms are much smaller in most usual cases and may be neglected.

As the sum of two trigonometric functions of one frequency is another trigonometric function with the same frequency, *ϵ* can be written as

(5) $$\begin{array}{cc} \epsilon (r,\lambda ) & =\epsilon_{0}^{\prime }\left(\lambda \right)+\epsilon_{1}^{\prime }\left(\lambda \right)\cos\left(K\cdot r+\Phi_{B}\right)\\ & +i\left(\epsilon_{0}^{\prime \prime }\left(\lambda \right)+\epsilon_{1}^{\prime \prime }\left(\lambda \right)\cos\left(K\cdot r+\Phi_{A}\right)\right) \end{array}$$

where

(6) $$\begin{array}{cc} \epsilon_{0}^{\prime }\left(\lambda \right) & ={n_{0}\left(\lambda \right)}^{2} \end{array}$$

(7) $$\begin{array}{cc} \epsilon_{0}^{\prime \prime }\left(\lambda \right) & =2{n_{0}\left(\lambda \right)}^{2}\kappa_{0}\left(\lambda \right) \end{array}$$

(8) $$\begin{array}{cc} \epsilon_{1}^{\prime }\left(\lambda \right) & =\sqrt{R_{B}{\left(\lambda \right)}^{2}+I_{B}{\left(\lambda \right)}^{2}} \end{array}$$

(9) $$\begin{array}{cc} \epsilon_{1}^{\prime \prime }\left(\lambda \right) & =\sqrt{R_{A}{\left(\lambda \right)}^{2}+I_{A}{\left(\lambda \right)}^{2}} \end{array}$$

(10) $$\begin{array}{cc} \Phi_{A}\left(\lambda \right) & =\pm \arccos\left(R_{A}\left(\lambda \right)/\epsilon_{1}^{\prime \prime }\left(\lambda \right)\right)\;\;\;\text{(positive}\;\text{if}\;R_{A}\left(\lambda \right)<0\text{)} \end{array}$$

(11) $$\begin{array}{cc} \Phi_{B}\left(\lambda \right) & =\pm \arccos\left(R_{B}\left(\lambda \right)/\epsilon_{1}^{\prime }\left(\lambda \right)\right)\;\;\;\text{(positive}\;\text{if}\;R_{B}\left(\lambda \right)<0\text{)} \end{array}$$

(12) $$\begin{array}{cc} R_{A}\left(\lambda \right) & =2n_{0}{\left(\lambda \right)}^{2}\kappa_{1}\left(\lambda \right)\cos\left(\Phi_{\kappa }\right)+4n_{0}\left(\lambda \right)n_{1}\left(\lambda \right)\kappa_{0}\left(\lambda \right)\cos\left(\Phi_{n}\right) \end{array}$$

(13) $$\begin{array}{cc} I_{A}\left(\lambda \right) & =2n_{0}{\left(\lambda \right)}^{2}\kappa_{1}\left(\lambda \right)\sin\left(\Phi_{\kappa }\right)+4n_{0}\left(\lambda \right)n_{1}\left(\lambda \right)\kappa_{0}\left(\lambda \right)\sin\left(\Phi_{n}\right) \end{array}$$

(14) $$\begin{array}{cc} R_{B}\left(\lambda \right) & =-2n_{0}{\left(\lambda \right)}^{2}\kappa_{0}\left(\lambda \right)\kappa_{1}\left(\lambda \right)\cos\left(\Phi_{\kappa }\right)+2n_{0}\left(\lambda \right)n_{1}\left(\lambda \right)\kappa_{0}\left(\lambda \right)\cos\left(\Phi_{n}\right) \end{array}$$

(15) $$\begin{array}{cc} I_{B}\left(\lambda \right) & =-2n_{0}{\left(\lambda \right)}^{2}\kappa_{0}\left(\lambda \right)\kappa_{1}\left(\lambda \right)\sin\left(\Phi_{\kappa }\right)+2n_{0}\left(\lambda \right)n_{1}\left(\lambda \right)\kappa_{0}\left(\lambda \right)\sin\left(\Phi_{n}\right) \end{array}$$

are the cumulated real and imaginary modulations of the permittivity and their phase shifts. Thus, a sinusoidal grating in *n* and *κ* is also a sinusoidal grating in *ϵ* in good approximation. In contrast to [17] and [7], however, we will proceed in our calculation using the permittivity.

## 3. Dispersion of First Order Diffracted Waves

### 3.1. Signal Wave Reconstruction

**Figure 2:**
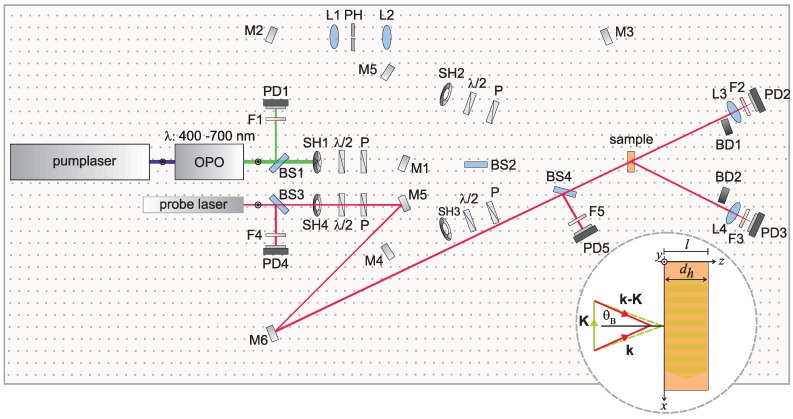
Sketch of an optical setup for the reconstruction of a test hologram: Closing of shutter SH1 stops the hologram recording process. Then, reconstruction with a wavelength different from the recording wavelength is enabled by opening shutter SH4. The direction of the probe beam, particularly the angle of incidence with respect to the samples’ normal, is adjusted according to momentum conservation depicted in the inset figure.

Upon the recording of the test hologram, the complex permittivity *ϵ* of the sample will be modulated along the *x*-direction. Assuming a linear recording mechanism, a sinusoidal modulation proportional to I(x) can be expected. Hence, this permittivity grating is also characterized by the wave vector K. Reconstruction of the signal wave S can be performed by exposure of K with a monochromatic wave (wavevector k, |k|=2π/λ and angle of incidence *θ* measured with respect to the hologram’s normal). Wavelength and angle of incidence can be chosen in a rather broad range provided that the complex permittivity allows for wave propagation in the medium and the permittivity modulation extends into the desired reconstruction wavelength range. The wavelength and the propagation direction, *i.e*., the wavevector of the reconstructed wave are unambiguously determined by momentum conservation, resulting in the wavevector $k_{S}=k_{R}-K$ (cf. Figure 2). We note here that for an arbitrarily chosen reconstruction wave $k_{R}$ in the majority of cases $|k_{R}\left|\neq \right|k_{S}|$ and the angle of the reference wave to the hologram’s normal will differ from the angle of the signal wave.

However, the appearance of wavelengths in the reconstructed beam spectrum different from the wavelength of the reconstructing wave is impossible, *i.e*., ($|k_{S}\left|=\right|k_{R}|$), if frequency conversion or inelastic scattering phenomena (e.g., Raman scattering) are not to be considered in the holographic medium under study. In this particular case, conservation of energy enforces a specific angle of incidence for a given wavelength of the reconstruction wave, commonly denoted as the *Bragg* angle $\theta_{B}$ and determined by $\theta_{B}=\arcsin\left(\lambda /2\Lambda \right)$. We note that the Bragg angle and the angle of recording coincide ($\theta_{B}=\theta$) solely for identical wavelengths of recording and reconstruction beams.

Conservation of energy and momentum defines a sharp constraint for the possibility of wave reconstruction for a given grating vector **K**. However, in any practical situation, this condition is softened by at least two effects, leading to the well known rocking curve (*i.e*., the shape of the signal wave’s intensity profile upon angular or wavelength detuning from the Bragg condition): 1. Every existing laser system has a certain wavelength bandwidth. Consequently, there is not only one writing wavelength *λ* but a range [λ−δλ,λ+δλ], which is resembled either by the range of possible reconstruction wavelengths under a certain angle of incidence or the range of angles for a given wavelength; 2. The sharpness of a grating is inversely proportional to the dimensions of the grating (cf. Figure 24.5a,b in [25]). In *z*-direction, the uncertainty of the Bragg condition of a grating with thickness *d* is given by 2π/d. Consequently, the Bragg condition is a property of thick holograms. In *x*-direction, the dependency on the dimensions is even more obvious, as for an extension of the illumination that is smaller than Λ, the modulation would lose its grating characteristics. As commonly used beam diameters typically illuminate several hundreds or thousands of grating periods, finiteness in *x*-direction can be neglected. The finiteness of the grating in *z*-direction, however, which is governed either by the thickness of the crystal or the effective penetration depth of the recording beam, is covered. The *z*-dependency will be considerable for common samples and may be used to determine the effective grating thickness $d_{\text{eff}}$.

The spectral width of the recording beam is assumed to be sharp compared with other effects in the theory, but related effects can be addressed via the spectroscopic methods presented in the following.

From the experimental point of view, this implies that reconstruction of the signal beam S can be simply performed by exposure of the recorded grating to the reference beam, *i.e*., by shutting the signal beam via shutter SH3. Then, the Bragg condition is automatically fulfilled. Alternatively, probing wavelengths different from the recording wavelength can be used provided the fulfilment of the Bragg condition via angular adjustment (The experimental values (wavelengths, angle of incidence) are determined outside the hologram media. In contrast, the theoretical derivation of the underlying wave coupling process proceeds for measures inside the sample. Hence, wavelengths and propagation angle of recording and probe beams in the experiment must be corrected for refraction.)

The basic setup for signal beam reconstruction exemplarily for a particular probing wavelength *λ* slightly larger than the recording wavelength is sketched in Figure 2.

Compared with Figure 1, shutters SH1 and SH4 have been closed and opened, respectively. Bragg-matched read-out is verified by choosing an appropriate angle of incidence. Furthermore, the hologram lifetime $\tau_{h}$ is much larger than the time required for reconstruction. The reconstructed signal beam is detected by its intensity using photodiode PD3, *i.e*., the presence of a hologram is verified by an intensity ${\left|S\right|}^{2}>0$. Its quality is defined via the diffraction efficiency $\eta_{1}={\left|S\right|}^{2}/{\left|R_{0}\right|}^{2}$, that is determined predominantly by the amplitude of change of the complex permittivity as shown in the following theoretical analysis.

With this setup in mind and the properties of the Bragg condition mentioned above, we are able to study the dispersive properties of the recorded grating by applying a tunable probe laser. Technically, the angular adjustment for each probing wavelength can be performed by different means. We here mention the application of a motorized linear or rotation stage for automatic adjustment of the reconstruction beam. But also the rotation of the hologram medium itself can be considered in the case of a sufficient hologram lifetime. White light generation using an ultrafast laser system has become very attractive for generation of a broad-band light intensity spectrum. Its divergence angle can be adjusted properly to match with the angular range of the Bragg condition.

The determination of grating parameters over a broad spectral range then can be used for an exhausting analysis of the photophysical effects that are underlying the hologram recording (*holographic spectroscopy*). It should be noted, that tuning the recording wavelength is also possible, as indicated by the tunable laser source applied for hologram recording in Figure 1. In this case, the dispersive properties of the photosensitive response are retrieved.

### 3.2. Coupled-Wave Theory

Reconstruction can be understood as light diffraction at a thick, periodic permittivity grating. We will construct a first order coupled-wave theory modulation according to the simplified sketch in the inset of Figure 1.

Introducing Equation (5) into Equation (1) results in a linear partial differential equation (PDE) with complex, periodic coefficients. The problem including the boundary conditions is isotropic in *y* direction and K-periodic in *x* direction, but still strongly depends on the *z* direction. Consequently, an ansatz for the solution of an incoming beam with wave vector $k=k_{0}{\left(\epsilon_{0}^{\prime }\left(\lambda \right)\right)}^{1/2}{\left(\sin\left(\theta \right),0,\cos\left(\theta \right)\right)}^{t}$ is provided by the Fourier series $E\left(r\right)=\sum_{n=-\infty }^{\infty }A_{n}\left(z\right)\exp\left(i(k-nK)\cdot r\right)$. The terms in the sum may be interpreted as diffracted beams with wave vector k−nK and complex amplitude $A_{n}\left(z\right)$. We will refer to them as the *n*-th diffraction orders.

By equating coefficients of the exponential functions, an infinite set of ordinary second order differential equations can be derived. The influence of the second order has been discussed in [26]. Due to the fact that it is small even in heavily modulated thick gratings, we will neglect it in the following.

The resulting ordinary differential equations (ODEs) have the form

(16) $$A_{n}^{\prime }\left(z\right)=aA_{n-1}\left(z\right)+b_{n}A_{n}\left(z\right)+cA_{n+1}\left(z\right)$$

where *a*, *b*, *c* are complex numbers. This coupling between the Fourier coefficients, *i.e*., the outgoing waves, emerges from the terms exp(iK·r) and exp(−iK·r) of the exponential representation of *ϵ* being multiplied with *E* and thus resulting in a term $A_{n}\left(z\right)\exp\left((k-(n\pm 1)K)\cdot r\right)$. Consequently, every equation of the complete infinite set is iteratively coupled to any other via the equations between.

In a diffraction experiment, only $A_{0}\left(0\right)\neq 0$ in the boundary conditions and, due to the iterative coupling, $A_{n}\left(z\right)\neq 0$, if and only if one of its neighbouring coefficients $A_{m}\left(z\right)\neq 0$ for m=n±1. Thus, the beam intensity can only build up “from the inside to the outside”, *i.e*., from lower to higher absolute diffraction orders.

The system (16) of coupled differential equations can be written in vector form as

(17) $$A^{\prime }\left(z\right)=\hat{B}A$$

where $\hat{B}$ is an infinite tridiagonal matrix. Equation (17) will have the formal solution $A\left(z\right)=\exp\left(\hat{B}z\right)A\left(0\right)$. In order to evaluate it one needs the eigenvalues and eigenvectors of $\hat{B}$. They could be determined numerically, or, because the entries of the secondary diagonals of B are typically three orders of magnitude smaller than its diagonal elements, by means of the perturbation theory. Then we are faced with the following problem: In the in-Bragg case ($\theta =\theta_{B}$) two diagonal elements of $\hat{B}$ coincide, namely $b_{1}=b_{0}$, and one has to invoke the twofold degenerate perturbation theory. However, in the far-off-Bragg case (e.g., $\theta \gg \theta_{B}$) the non-degenerate perturbation theory has to be applied. Both theories give different results and the need for a unified approximation that can be applied in the whole domain arises. It turns out that the two-coupled wave approximation (Kogelnik ansatz) can be viewed as an interpolation between the results of perturbation theory in the two extremal cases in-Bragg and far-off-Bragg, as far as the two waves $A_{0}\left(z\right)$ and $A_{1}\left(z\right)$ are concerned. The details of this argument will be published elsewhere. Here we take these remarks only as an additional justification to follow the Kogelnik ansatz for the diffraction problem.

Instead, this purely mathematical approach may be greatly simplified by taking into account the physical nature of the problem. As explained above, due to momentum and energy conservation the Bragg condition, allows for only up to one signal beam. The previous considerations demand this beam to be described by $A_{\pm 1}\left(z\right)$: Depending on the sign of the angle of incidence and the choice of the direction of *K*, $A_{\pm 1}\left(z\right)\exp\left(i(k-K)\cdot r\right)$ is thus the only possible wave satisfying the Bragg condition for the incident beam $A_{0}\left(z\right)\exp\left(i(k)\cdot r\right)$. All other parts of the solution are assumed to be damped exponentially and do thus not develop notably. It has been shown [27,28] that these higher diffractions do not play a role even for high diffraction efficiencies for incidence angles below $45^{\circ }$. The damping effect even for minor deviations of the remaining reference beam from the Bragg angle will be shown in the off-Bragg case, which will be discussed later.

Furthermore, this constitutes our additional argument to omit the second class of nonlinear terms in Section 2, as any contribution proportional to $\cos\left(nK\cdot r+\phi \right)$, |n|>1, would couple only into the higher orders of the Fourier series shown above and thus induce a beam violating the Bragg condition. (They would, however define an additional Bragg angle, but are neglected due to the considerable lower diffraction efficiency. This fact is especially important for periodic gratings that deviate from the sinusoidal form, such as gratings that are excited up to the saturation region [29]. At least for smaller deviations, the present theory may still be applied, only using the first order of their Fourier series, as any other order couples in beams violating the Bragg condition.)

These considerations result in a two-wave ansatz

(18) $$E\left(r\right)=R\left(z\right)\exp\left(ik\cdot r\right)+S\left(z\right)\exp\left(i(k\pm K)\cdot r\right)$$

where $R\left(z\right)=A_{0}\left(z\right)$ is the incident reference beam, $S\left(z\right)=A_{1}\left(z\right)$ is the diffracted signal beam and again, the sign depends on the angle of incidence and the choice of the direction of *K*. Compared with the ansatz by Kogelnik, which uses the negative sign, this version allows to take into account the effects of non-phase-symmetrical gratings, *i.e*., gratings with Φ≠0 or Φ≠π, which are not invariant against a reflection about the z-axis. We will, however, proceed with the negative sign, which is valid for a grating vector and beam incidence in (partially) positive *x* direction (θ>0), and integrate the incidence for θ<0 via the symmetry argument θ→−θ⇒Φ→−Φ.

We note that a solution taking into account higher orders of diffraction (Rigorous Coupled-Wave Theory) has been presented in [8,30,31,32]. A comparison with Kogelnik’s theory has recently been given in [33]. These orders are especially important for thin gratings, where the damping effect of the violation of the Bragg conditions is less relevant. The off-Bragg directional performance of this choice for a k-vector, the beta-value method (BVM) published in [9] has been studied experimentally in comparison with the Kogelnik ansatz in [34].

Inserting Equation (18) into Equation (1) and substituting $\alpha_{0}=2k_{0}{\left(\epsilon_{0}^{\prime }\left(\lambda \right)\right)}^{1/2}\kappa_{0}$ gives the following two coupled-wave equations:

(19) $$\begin{array}{c} \begin{array}{cc} \frac{\text{d}R(z)}{\text{d}z} & =-\frac{\alpha_{0}}{2\cos(\theta )}R\left(z\right)\\ & -\frac{e^{i\Phi_{A}}k_{0}\epsilon_{1}^{\prime \prime }\left(\lambda \right)S\left(z\right)}{4{\left(\epsilon_{0}^{\prime }\left(\lambda \right)\right)}^{1/2}\cos\left(\theta \right)}+\frac{ie^{i\Phi_{B}}k_{0}\epsilon_{1}^{\prime }\left(\lambda \right)S\left(z\right)}{4{\left(\epsilon_{0}^{\prime }\left(\lambda \right)\right)}^{1/2}\cos\left(\theta \right)} \end{array} \end{array}$$

(20) $$\begin{array}{c} \begin{array}{cc} \frac{\text{d}S(z)}{\text{d}z} & =-\frac{\alpha_{0}}{2\cos(\theta )}S\left(z\right)\\ & -\frac{e^{-i\Phi_{A}}k_{0}\epsilon_{1}^{\prime \prime }\left(\lambda \right)R\left(z\right)}{4{\left(\epsilon_{0}^{\prime }\left(\lambda \right)\right)}^{1/2}\cos\left(\theta \right)}+\frac{ie^{-i\Phi_{B}}k_{0}\epsilon_{1}^{\prime }\left(\lambda \right)R\left(z\right)}{4{\left(\epsilon_{0}^{\prime }\left(\lambda \right)\right)}^{1/2}\cos\left(\theta \right)}\\ & +i\left(\frac{K_{x}^{2}}{2k_{0}{\left(\epsilon_{0}^{\prime }\left(\lambda \right)\right)}^{1/2}\cos\left(\theta \right)}-K_{x}\tan\left(\theta \right)\right)S\left(z\right) \end{array} \end{array}$$

As explained in the initial table, the illumination is assumed to be constant in *y*-direction and infinitely large. Thus the equation does not depend on *y*.

In the limit case of a zero grating modulation $\epsilon_{1}^{\prime }=\epsilon_{1}^{\prime \prime }=0$, the first term of each equation, *i.e*., the diagonal of the differential equation system, remains and gives the well known Beer–Lambert law for the base absorption in the material. With non-zero grating modulation, a Beer–Lambert-like exponential decay is maintained, but it is governed by the mean absorption of the grating. It then contributes to the different decay characteristics of the transmitted and diffracted beams. The diagonal can be eliminated in the following by introducing the law into the ansatz using the substitution $R\left(z\right)=\bar{R}\left(z\right)\exp(-\alpha_{0}z/\left(2\cos\left(\theta \right)\right)$ and $S\left(z\right)=\bar{S}\left(z\right)\exp(-\alpha_{0}z/\left(2\cos\left(\theta \right)\right)$, resulting in the equations

(21) $$\begin{array}{c} \begin{array}{cc} \frac{\text{d}\bar{R}\left(z\right)}{\text{d}z} & =\frac{e^{i\Phi_{A}}k_{0}\epsilon_{1}^{\prime \prime }\left(\lambda \right)\bar{S}\left(z\right)}{4{\left(\epsilon_{0}^{\prime }\left(\lambda \right)\right)}^{1/2}\cos\left(\theta \right)}-\frac{ie^{i\Phi_{B}}k_{0}\epsilon_{1}^{\prime }\left(\lambda \right)\bar{S}\left(z\right)}{4{\left(\epsilon_{0}^{\prime }\left(\lambda \right)\right)}^{1/2}\cos\left(\theta \right)} \end{array} \end{array}$$

(22) $$\begin{array}{c} \begin{array}{cc} \frac{\text{d}\bar{S}\left(z\right)}{\text{d}z} & =\frac{e^{-i\Phi_{A}}k_{0}\epsilon_{1}^{\prime \prime }\left(\lambda \right)\bar{R}\left(z\right)}{4{\left(\epsilon_{0}^{\prime }\left(\lambda \right)\right)}^{1/2}\cos\left(\theta \right)}-\frac{ie^{-i\Phi_{B}}k_{0}\epsilon_{1}^{\prime }\left(\lambda \right)\bar{R}\left(z\right)}{4{\left(\epsilon_{0}^{\prime }\left(\lambda \right)\right)}^{1/2}\cos\left(\theta \right)}\\ & +i\left(\frac{K_{x}^{2}}{2k_{0}{\left(\epsilon_{0}^{\prime }\left(\lambda \right)\right)}^{1/2}\cos\left(\theta \right)}-K_{x}\tan\left(\theta \right)\right)\bar{S}\left(z\right) \end{array} \end{array}$$

Here and in the following, the barred variables represent quantities adjusted by the mean absorption.

In this form, Equation (21) is reduced solely to the coupling, as the two terms are governed by the amplitude of the other beam, S(z). Equation (22) shows similar coupling terms, but also feature an additional term in the last line of Equation (22), becoming zero in the case that $\theta \;=\;\theta_{B}=\;\arcsin\left(K_{x}/\left(2k_{0}{\left(\epsilon_{0}^{\prime }\left(\lambda \right)\right)}^{1/2}\right)\right)$, *i.e*., if the Bragg condition is fulfilled. Introducing this formula into the last line, *i.e*., expressing $K_{x}$ through $\theta_{B}$, results in a coefficient

(23) $${\left(k_{0}\epsilon_{0}^{\prime }\left(\lambda \right)\right)}^{1/2}\left(\frac{2\sin{\left(\theta_{B}\right)}^{2}}{\cos(\theta )}-2\sin\left(\theta_{B}\right)\tan\left(\theta \right)\right)$$

From this, we define the off-Bragg parameter as

(24) $$\beta \left(\theta \right)=\frac{2\sin{\left(\theta_{B}\right)}^{2}}{\cos(\theta )}-2\sin\left(\theta_{B}\right)\tan\left(\theta \right)$$

The Taylor series given in the second line of Equation (24) is fairly accurate over the range of most rocking curves even for large Bragg angles. Thus, for calculations in the vicinity of the Bragg angle, the approximation

(25) $$\beta \left(\theta \right)\approx 2\left(\theta_{B}-\theta \right)\sin\left(\theta_{B}\right)$$

may be used.

### 3.3. Diffraction Efficiency of Out-of-Phase Mixed Gratings

For an analytical examination, we express Equations (21) and (22) in vector form. The coefficient matrix of the resulting ODE is

(26) $$\hat{A}=\left(\begin{array}{cc} i\beta \left(\theta \right)k_{0}{\left(\epsilon_{0}^{\prime }\left(\lambda \right)\right)}^{1/2} & \kappa_{-}\\ \kappa_{+} & 0 \end{array}\right)$$

with

(27) $$\kappa_{\pm }=-\frac{k_{0}\left(\exp\left(i\pm \Phi_{A}\right)\epsilon_{1}^{\prime \prime }\left(\lambda \right)-i\exp\left(i\pm \Phi_{B}\right)\epsilon_{1}^{\prime }\left(\lambda \right)\right)}{4{\left(\epsilon_{0}^{\prime }\left(\lambda \right)\right)}^{1/2}\cos\left(\theta \right)}$$

This form of the coupling coefficients exhibits a much higher degree of symmetry than the ones used in other publications. One reason for this is the use of one complex quantity, *i.e*., the permittivity, instead of the two real quantities refractive index and absorption. Indeed, this allows for a considerably better algebraic simplification of the results obtained from the differential equations. Therefore, we will not need to use the abbreviations $\kappa_{\pm }$ in the following.

In a diffraction experiment, there is no natural choice for x=0, as there is no initial interference pattern. Consequently, one of the phase offsets of the gratings can be chosen as 0 without loss of generality. Thus, only the difference between $\Phi_{A}$ and $\Phi_{B}$ matters and the phase difference $\phi_{\text{Diff}}\left(z\right)$ between the reference and the signal wave establishes itself accordingly. In the following, we choose $\Phi_{B}=0$ and $\Phi_{A}=\Phi$.

The general solution of the problem is given by

(28) $$\left(\begin{array}{c} \bar{R}\\ \bar{S} \end{array}\right)=C_{1}A_{1}e^{a_{1}z}+C_{2}A_{2}e^{a_{2}z}$$

where $A_{1,2}$ are the eigenvectors of $\hat{A}$ and $a_{1,2}$ are their related eigenvalues. The coefficients $C_{1,2}$ emerge from the boundary conditions, which in the most general case with a normalized reference intensity are $\bar{R}\left(0\right)=1$, $\bar{S}\left(0\right)=S\left(0\right)/\left|R\left(0\right)\right|$, whereby S(0)=0 in a diffraction experiment. Both the eigensystem and the equation emerging from the boundary conditions can be solved via standard algebraic methods without approximations. Using the boundary condition S(0)=0, the differential equation solved to

(29) $$\begin{array}{cc} \bar{R}\left(z,\theta \right) & =e^{\frac{1}{2}i\beta \left(|\theta |\right){\left(\epsilon_{0}^{\prime }\left(\lambda \right)\right)}^{1/2}zk_{0}}\\ & (\cos\left(\frac{zk_{0}\sqrt{{\left(2\beta \left(|\theta |\right)\cos\left(\theta \right)\epsilon_{0}^{\prime }\left(\lambda \right)\right)}^{2}-\epsilon_{1}^{\prime \prime }{\left(\lambda \right)}^{2}+2i\cos\left(\Phi \right)\epsilon_{1}^{\prime \prime }\left(\lambda \right)\epsilon_{1}^{\prime }\left(\lambda \right)+\epsilon_{1}^{\prime }{\left(\lambda \right)}^{2}}}{4{\left(\epsilon_{0}^{\prime }\left(\lambda \right)\right)}^{1/2}\cos\left(\theta \right)}\right)\\ & -\frac{i(2\beta (|\theta |)\cos\left(\theta \right)\epsilon_{0}^{\prime }\left(\lambda \right))\sin\left(\frac{zk_{0}\sqrt{{\left(2\beta \left(|\theta |\right)\cos\left(\theta \right)\epsilon_{0}^{\prime }\left(\lambda \right)\right)}^{2}-\epsilon_{1}^{\prime \prime }{\left(\lambda \right)}^{2}+2i\cos\left(\Phi \right)\epsilon_{1}^{\prime \prime }\left(\lambda \right)\epsilon_{1}^{\prime }\left(\lambda \right)+\epsilon_{1}^{\prime }{\left(\lambda \right)}^{2}}}{4{\left(\epsilon_{0}^{\prime }\left(\lambda \right)\right)}^{1/2}\cos\left(\theta \right)}\right)}{\sqrt{{\left(2\beta \left(|\theta |\right)\cos\left(\theta \right)\epsilon_{0}^{\prime }\left(\lambda \right)\right)}^{2}-\epsilon_{1}^{\prime \prime }{\left(\lambda \right)}^{2}+2i\cos\left(\Phi \right)\epsilon_{1}^{\prime \prime }\left(\lambda \right)\epsilon_{1}^{\prime }\left(\lambda \right)+\epsilon_{1}^{\prime }{\left(\lambda \right)}^{2}}}) \end{array}$$

and

(30) $$\begin{array}{cc} \bar{S}\left(z,\theta \right) & =ie^{\frac{1}{2}i\beta \left(|\theta |\right){\left(\epsilon_{0}^{\prime }\left(\lambda \right)\right)}^{1/2}zk_{0}}\left(\epsilon_{1}^{\prime }\left(\lambda \right)+ie^{-i\text{sgn}(\theta )\Phi }\epsilon_{1}^{\prime \prime }\left(\lambda \right)\right)\;\\ & \frac{\sin\left(\frac{\sqrt{{\left(2\beta \left(|\theta |\right)\cos\left(\theta \right)\epsilon_{0}^{\prime }\left(\lambda \right)\right)}^{2}-\epsilon_{1}^{\prime \prime }{\left(\lambda \right)}^{2}+2i\cos\left(\Phi \right)\epsilon_{1}^{\prime \prime }\left(\lambda \right)\epsilon_{1}^{\prime }\left(\lambda \right)+\epsilon_{1}^{\prime }{\left(\lambda \right)}^{2}}k_{0}z}{4{\left(\epsilon_{0}^{\prime }\left(\lambda \right)\right)}^{1/2}\cos\left(\theta \right)}\right)}{\sqrt{{\left(2\beta \left(|\theta |\right)\cos\left(\theta \right)\epsilon_{0}^{\prime }\left(\lambda \right)\right)}^{2}-\epsilon_{1}^{\prime \prime }{\left(\lambda \right)}^{2}+2i\cos\left(\Phi \right)\epsilon_{1}^{\prime \prime }\left(\lambda \right)\epsilon_{1}^{\prime }\left(\lambda \right)+\epsilon_{1}^{\prime }{\left(\lambda \right)}^{2}}} \end{array}$$

Here, we have already introduced the symmetry argument, manifesting in the form the absolute value of *θ* and the sign function in front of Φ.

The diffraction efficiency, still assuming $\bar{S}\left(0\right)=0$, is given by

(31) $$\eta_{1}=\frac{I_{S}\left(z\right)}{I_{R}\left(0\right)}=A_{LB}\left(\theta \right){\left|\frac{\bar{S}\left(z\right)}{\bar{R}\left(0\right)}\right|}^{2}$$

where $A_{LB}\left(\theta \right)=\exp\left(-\alpha_{0}z/\left(2\cos(\theta )\right)\right)$ is the ground absorption from the Beer–Lambert law.

Note again that $\alpha_{0}$ is the mean absorption coefficient of the grating and does in general not equal the absorption coefficient of the crystal before the grating is written. This is particularly important when a writing pattern does not penetrate the full depth of the crystal and the absorption behind the grating has to be added to the calculation of the result (see e.g., discussion in [5]). Application of the above results leads to the general equation for arbitrary mixed out-of-phase gratings.

(32) $$\begin{array}{cc} \eta_{1}\left(z,\theta \right) & =A_{\text{LB}}\left(\theta \right){\left|\epsilon_{1}^{\prime }\left(\lambda \right)+ie^{\text{sgn}(\theta )i\Phi }\epsilon_{1}^{\prime \prime }\left(\lambda \right)\right|}^{2}\\ & \frac{{\left|\sin\left(\frac{\sqrt{{\left(2\beta \left(|\theta |\right)\cos\left(\theta \right)\epsilon_{0}^{\prime }\left(\lambda \right)\right)}^{2}-\epsilon_{1}^{\prime \prime }{\left(\lambda \right)}^{2}+2i\cos\left(\Phi \right)\epsilon_{1}^{\prime \prime }\left(\lambda \right)\epsilon_{1}^{\prime }\left(\lambda \right)+\epsilon_{1}^{\prime }{\left(\lambda \right)}^{2}}k_{0}z}{4{\left(\epsilon_{0}^{\prime }\left(\lambda \right)\right)}^{1/2}\cos\left(\theta \right)}\right)\right|}^{2}}{\left|{\left(2\beta \left(|\theta |\right)\cos\left(\theta \right)\epsilon_{0}^{\prime }\left(\lambda \right)\right)}^{2}-\epsilon_{1}^{\prime \prime }{\left(\lambda \right)}^{2}+2i\cos\left(\Phi \right)\epsilon_{1}^{\prime \prime }\left(\lambda \right)\epsilon_{1}^{\prime }\left(\lambda \right)+\epsilon_{1}^{\prime }{\left(\lambda \right)}^{2}\right|} \end{array}$$

As this equation depends on all three grating parameters, $\epsilon_{1}^{\prime }$, $\epsilon_{1}^{\prime \prime }$ and Φ, a comprehensive plot is not possible. We will discuss various aspects and limit cases in the following.

First, note that in this representation only the second factor depends on the sign of the grating phase, as it only appears as the argument of even functions in the fraction. The argument of the square root and the absolute value in the denominator, $(2\beta (|\theta |)\cos\left(\theta \right)\epsilon_{0}^{\prime }{\left(\lambda \right))}^{2}-\epsilon_{1}^{\prime \prime }{\left(\lambda \right)}^{2}+2i\cos\left(\Phi \right)\epsilon_{1}^{\prime \prime }\left(\lambda \right)\epsilon_{1}^{\prime }\left(\lambda \right)+\epsilon_{1}^{\prime }{\left(\lambda \right)}^{2}$, is real for any pure grating ($n_{1}=0$ or $\alpha_{1}=0$) or for gratings with Φ=±π/2. In the latter case, it is also positive under the condition $\epsilon_{1}^{\prime \prime }\left(\lambda \right)\leq \epsilon_{1}^{\prime }\left(\lambda \right)$. The only complex element in S(z) then is a global factor $\exp(2i\epsilon_{0}^{\prime }\left(\lambda \right)\beta \left(|\theta |\right)k_{0}z)$, resembling a phase shift linear to the off-Bragg factor β(|θ|). In out-of-phase gratings, however, the term $2i\cos\left(\Phi \right)\epsilon_{1}^{\prime \prime }\left(\lambda \right)\epsilon_{1}^{\prime }\left(\lambda \right)$ adds an imaginary component.

### 3.4. Pure Refractive Index and Absorption Gratings

Pure gratings are a special case of in-phase gratings. In the case of a pure refraction grating ($\alpha_{1}=0$), the diffraction efficiency reduces to

(33) $$\begin{array}{cc} \eta_{1}\left(z,\theta \right) & =A_{\text{LB}}\left(\theta \right)\epsilon_{1}^{\prime }{\left(\lambda \right)}^{2}\frac{\sin{\left(\frac{\sqrt{\epsilon_{1}^{\prime }{\left(\lambda \right)}^{2}+{\left(2\beta \left(|\theta |\right)\cos\left(\theta \right)\epsilon_{0}^{\prime }\left(\lambda \right)\right)}^{2}}k_{0}z}{4{\left(\epsilon_{0}^{\prime }\left(\lambda \right)\right)}^{1/2}\cos\left(\theta \right)}\right)}^{2}}{{\left(2\beta \left(|\theta |\right)\cos\left(\theta \right)\epsilon_{0}^{\prime }\left(\lambda \right)\right)}^{2}+\epsilon_{1}^{\prime }{\left(\lambda \right)}^{2}} \end{array}$$

and, in case of a pure absorption grating ($n_{1}=0$), to

(34) $$\eta_{1}\left(z,\theta \right)=A_{\text{LB}}\left(\theta \right)\epsilon_{1}^{\prime \prime }{\left(\lambda \right)}^{2}\frac{{\left|\sinh\left(\frac{\sqrt{\epsilon_{1}^{\prime \prime }{\left(\lambda \right)}^{2}-{\left(2\beta \left(|\theta |\right)\cos\left(\theta \right)\epsilon_{0}^{\prime }\left(\lambda \right)\right)}^{2}}k_{0}z}{4{\left(\epsilon_{0}^{\prime }\left(\lambda \right)\right)}^{1/2}\cos\left(\theta \right)}\right)\right|}^{2}}{\left|{\left(2\beta \left(|\theta |\right)\cos\left(\theta \right)\epsilon_{0}^{\prime }\left(\lambda \right)\right)}^{2}-\epsilon_{1}^{\prime \prime }{\left(\lambda \right)}^{2}\right|}$$

The hyperbolic sine in Equation (34) is achieved by extracting *i* from the square root according to |sin(ix)|=|sinh(x)|, resulting in the well-known forms for pure gratings. Notice, however, that thinking in terms of a hyperbolic *z* dependency of Equation (34) does only make sense in the vicinity of the Bragg angle as the square root will be imaginary again for larger β(|θ|).

### 3.5. In-Bragg Cases

In the in-Bragg case, Equation (32) simplifies to

(35) $$\begin{array}{cc} \eta_{1}\left(z,\theta_{B}\right)= & A_{\text{LB}}\left(\theta_{B}\right)\frac{\epsilon_{1}^{\prime \prime }{\left(\lambda \right)}^{2}+2\text{sgn}\left(\theta \right)\sin\left(\Phi \right)\epsilon_{1}^{\prime \prime }\left(\lambda \right)\epsilon_{1}^{\prime }\left(\lambda \right)+\epsilon_{1}^{\prime }{\left(\lambda \right)}^{2}}{\sqrt{\epsilon_{1}^{\prime \prime }{\left(\lambda \right)}^{4}+2\cos\left(2\Phi \right)\epsilon_{1}^{\prime \prime }{\left(\lambda \right)}^{2}\epsilon_{1}^{\prime }{\left(\lambda \right)}^{2}+\epsilon_{1}^{\prime }{\left(\lambda \right)}^{4}}}\cdot \\ & {\left|\sin\left(\frac{\sqrt{\epsilon_{1}^{\prime }{\left(\lambda \right)}^{2}+2i\cos\left(\Phi \right)\epsilon_{1}^{\prime \prime }\left(\lambda \right)\epsilon_{1}^{\prime }\left(\lambda \right)-\epsilon_{1}^{\prime \prime }{\left(\lambda \right)}^{2}}k_{0}z}{4{\left(\epsilon_{0}^{\prime }\left(\lambda \right)\right)}^{1/2}\cos\left(\theta \right)}\right)\right|}^{2} \end{array}$$

In pure gratings the coefficient outside the modulo cancels down, resulting in the in-Bragg expressions

(36) $$\begin{array}{cc} \eta_{1}\left(z,\theta_{B}\right) & =A_{\text{LB}}\left(\theta \right)\sin{\left(\frac{\epsilon_{1}^{\prime }\left(\lambda \right)k_{0}z}{4{\left(\epsilon_{0}^{\prime }\left(\lambda \right)\right)}^{1/2}\cos\left(\theta \right)}\right)}^{2}\\ & =A_{\text{LB}}\left(\theta \right)\sin{\left(\frac{n_{1}\left(\lambda \right)k_{0}z}{2\cos(\theta )}\right)}^{2} \end{array}$$

and

(37) $$\begin{array}{cc} \eta_{1}\left(z,\theta_{B}\right) & =A_{\text{LB}}\left(\theta \right){\left|\sinh\left(\frac{\epsilon_{1}^{\prime \prime }\left(\lambda \right)k_{0}z}{4{\left(\epsilon_{0}^{\prime }\left(\lambda \right)\right)}^{1/2}\cos\left(\theta \right)}\right)\right|}^{2}\\ & =A_{\text{LB}}\left(\theta \right){\left|\sinh\left(\frac{n_{0}\left(\lambda \right)\kappa_{1}\left(\lambda \right)k_{0}z}{2\cos(\theta )}\right)\right|}^{2}\\ & =A_{\text{LB}}\left(\theta \right){\left|\sinh\left(\frac{\alpha_{1}z}{4\cos(\theta )}\right)\right|}^{2} \end{array}$$

The last lines in both cases resemble the results in [17].

Note that we have neglected the real permittivity modulation induced by an absorption index modulation and the imaginary modulation induced by a refractive index modulation discussed in Section 2, as these are considerably smaller than the main contribution in common setups. Then,

(38) $$\epsilon_{1}^{\prime }\left(\lambda \right)=2n_{0}n_{1}\;\text{and}\;\epsilon_{1}^{\prime \prime }\left(\lambda \right)=\frac{n_{0}\alpha_{1}}{k_{0}}$$

respectively.

In agreement with Kogelnik’s work, the maximal diffraction efficiency of a pure absorption grating is 1/27 or about 3.7%. This value is achieved in the Bragg angle for $\alpha_{0}=\alpha_{1}$ and $\alpha_{0}z/\left(4\cos\left(\theta_{B}\right)\right)=\arccos\left(2/\sqrt{3}\right)\approx 0.55$. In a pure refractive grating with $\alpha_{0}=0$, the maximal diffraction efficiency is 100%, obviously.

### 3.6. Transmission Efficiency

The general expression for the 0-th order (transmission) efficiency $\eta_{0}=\left|R\left(z\right)/R\left(0\right)\right|$, including off-Bragg, is slightly more extensive, consisting of a sum of two terms:

(39) $$\begin{array}{c} \eta_{0}=A_{\text{LB}}\left(\theta \right)|\cos\left(\frac{zk_{0}\sqrt{{\left(2\beta \left(|\theta |\right)\cos\left(\theta \right)\epsilon_{0}^{\prime }\left(\lambda \right)\right)}^{2}-\epsilon_{1}^{\prime \prime }{\left(\lambda \right)}^{2}+2i\cos\left(\Phi \right)\epsilon_{1}^{\prime \prime }\left(\lambda \right)\epsilon_{1}^{\prime }\left(\lambda \right)+\epsilon_{1}^{\prime }{\left(\lambda \right)}^{2}}}{4{\left(\epsilon_{0}^{\prime }\left(\lambda \right)\right)}^{1/2}\cos\left(\theta \right)}\right)\\ -\frac{i(2\beta (|\theta |)\cos\left(\theta \right)\epsilon_{0}^{\prime }\left(\lambda \right))\sin\left(\frac{zk_{0}\sqrt{{\left(2\beta \left(|\theta |\right)\cos\left(\theta \right)\epsilon_{0}^{\prime }\left(\lambda \right)\right)}^{2}-\epsilon_{1}^{\prime \prime }{\left(\lambda \right)}^{2}+2i\cos\left(\Phi \right)\epsilon_{1}^{\prime \prime }\left(\lambda \right)\epsilon_{1}^{\prime }\left(\lambda \right)+\epsilon_{1}^{\prime }{\left(\lambda \right)}^{2}}}{4{\left(\epsilon_{0}^{\prime }\left(\lambda \right)\right)}^{1/2}\cos\left(\theta \right)}\right)}{\sqrt{{\left(2\beta \left(|\theta |\right)\cos\left(\theta \right)\epsilon_{0}^{\prime }\left(\lambda \right)\right)}^{2}-\epsilon_{1}^{\prime \prime }{\left(\lambda \right)}^{2}+2i\cos\left(\Phi \right)\epsilon_{1}^{\prime \prime }\left(\lambda \right)\epsilon_{1}^{\prime }\left(\lambda \right)+\epsilon_{1}^{\prime }{\left(\lambda \right)}^{2}}}{|}^{2} \end{array}$$

In the general in-Bragg case, this reduces to

(40) $$\begin{array}{c} \eta_{0}=A_{\text{LB}}\left(\theta \right)|\cos\left(\frac{zk_{0}\sqrt{\epsilon_{1}^{\prime }{\left(\lambda \right)}^{2}+2i\cos\left(\Phi \right)\epsilon_{1}^{\prime \prime }\left(\lambda \right)\epsilon_{1}^{\prime }\left(\lambda \right)-\epsilon_{1}^{\prime }{\left(\lambda \right)}^{2}}}{4{\left(\epsilon_{0}^{\prime }\left(\lambda \right)\right)}^{1/2}\cos\left(\theta \right)}\right){|}^{2} \end{array}$$

For a pure refractive grating with $\alpha_{0}=0$, $\eta_{0}+\eta_{1}=1$ as expected.

## 4. Examples

Pure gratings have been extensively discussed in literature for the last 40+ years since the previously mentioned groundbreaking paper by Kogelnik. In the following, we will discuss two more complex cases, which have come into the focus of attention only in the last few years, due to the progress made in the fields of advanced materials and tunable lasers.

### 4.1. Spectroscopic in-Bragg Analysis of the Diffraction Efficiency

Usually, the diffraction efficiency is measured for a single wavelength of an angular range around the Bragg angle. The resulting curve is called rocking curve and examples are shown and discussed in the next section. Here, we will examine the dependency of the diffraction efficiency of mixed gratings of the reference-beam wavelength over a broad optical spectrum range.

The description of the complex permittivity related to a recorded hologram considers both the ground state permittivity and the optically induced change of the permittivity. The ground state permittivity is decisive for the overall transmittance of the recording and reconstruction waves. Particularly, it determines the efficiency of hologram reconstruction which is its key measure.

With respect to dispersive measurements of the efficiency, the ground state permittivity thus needs to be taken into account. Otherwise, misinterpretation of the efficiency may occur, *i.e*., losses of the efficiency as a function of the wavelength due to an increase of the mean imaginary part of the permittivity may be understood as a reduction of the amplitude of the permittivity change.

In the following, we will show our results on two exemplary systems, both modelled on lithium niobate. The base absorption coefficient and refractive index are modelled by using a simple resonator model. It results in a pronounced absorption band at the lower edge of the optical spectrum, *i.e*., at high photon energies, with a band edge ($\alpha =20\;{\mathrm{cm}}^{-1}$) at 300 nm for lithium niobate grown from the congruently melting composition. Exposure to light with photon energies in the vicinity of the band edge energy can be applied for the recording of holograms via the mechanism of interband photorefraction [35]. The tail of this absorption further determines the fundamental absorption in the visible and near-infrared spectrum, and is important for near-infrared recording mechanisms of holograms in lithium niobate [36].

**Figure 3:**
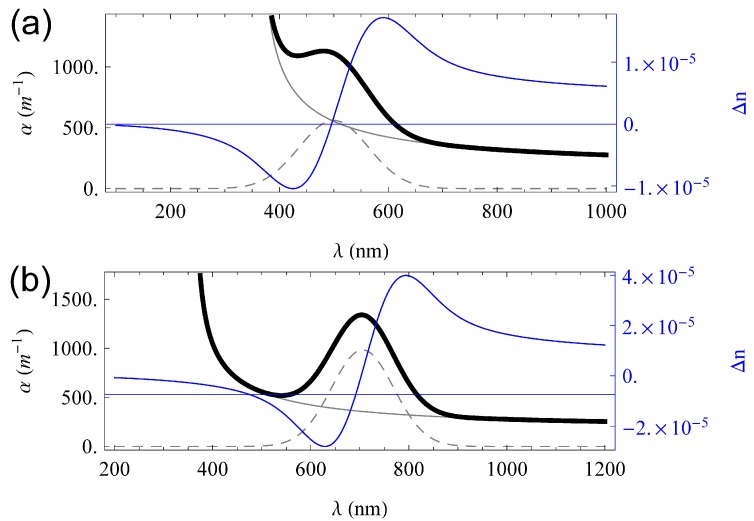
Full absorption coefficient (thick black), base absorption (grey black), modulated absorption band (dashed black) and refractive index change emerging from Kramers–Kronig relation (blue) for Gaussian absorption band related to Fe-doping (**a**) and polaronic absorption (**b**).

We now add a Gaussian-shaped absorption spectrum around 500nm resembling the absorption band of ${\mathrm{Fe}}^{2+}$ in Fe-doped lithium niobate and reveal the spectrum depicted in Figure 3a. Alternatively, we model a broad and asymmetric absorption spectrum of the form

(41) $$\alpha \left(\lambda \right)\propto \lambda \exp\left(-\frac{{\left(2E_{p}-\hbar c/\lambda \right)}^{2}}{4E_{p}\hbar \omega_{0}}\right)$$

according to the theoretical description of optical features of small bound polarons. This type of absorption, shown in Figure 3b exemplarily for the small bound ${\mathrm{Nb}}_{\mathrm{Li}}^{4+}$ electron polaron centered at 1.6 eV, describes the light-induced small polaron transfer from a specific lattice site to one of its next-neighbouring site. (For the given example, from a ${\mathrm{Nb}}_{\mathrm{Li}}^{4+}$ lattice site to one of the eight ${\mathrm{Nb}}_{\mathrm{Nb}}^{5+}$ sites, to which the defect is octahedrally coordinated.) Thus, the absorption is characterized by a minimum energy $E_{p}$ and a representative phonon energy of the system $\hbar \omega_{0}$ [37]. Here, *c* denotes the speed of light in vacuum and $\omega_{0}$ is the phonon frequency.

Contrary to the dominating oscillator for the band-to-band transition, the strengths of the ${\mathrm{Fe}}^{2+}$ and small polaron absorption characteristics are strongly affected by light-exposure: The electron is excited from ${\mathrm{Fe}}^{2+}$ to the valence band, thus ${\mathrm{Fe}}^{3+}$ remains, featuring an extremely sharp absorption band [38]. The light-induced optical transfer of the electron from the ${\mathrm{Nb}}_{\mathrm{Li}}^{4+}$ polaronic site to its neighbouring ${\mathrm{Nb}}_{\mathrm{Nb}}^{5+}$ site results in the disappearance of the near-infrared band at 1.6 eV, while an absorption band of the small free ${\mathrm{Nb}}_{\mathrm{Nb}}^{4+}$ electron polaron builds up at approximately 1.0 eV.

Taking into account these optically induced alterations of the absorption bands and the fact that potentially both bands can diminish completely, we can model the causally related light-induced change of the refractive index via Kramers–Kronig relation. Figure 3 shows the dispersion of the optically induced index amplitude $n_{1}$ free of the dispersion of the index $n_{0}\left(\lambda \right)$. In comparison with the classical recording of phase holograms via the photorefractive effect [39], we note that the dispersion of the index amplitude features a change of the sign within the optical spectrum so that spectra with both positive and negative dispersion appear. This feature has been experimentally verified for small-polaron recording of holograms in lithium niobate in [40].

For in-Bragg measurements, the Bragg angle has to be adjusted for each wavelength. The Bragg angle transforms according to

(42) $$\theta_{B}\left(\lambda \right)=\arcsin\left(\frac{\lambda {\left(\epsilon_{0}^{\prime }\left(\lambda_{R}\right)\right)}^{1/2}}{\lambda_{R}{\left(\epsilon_{0}^{\prime }\left(\lambda \right)\right)}^{1/2}}\sin\left(\theta_{B,R}\right)\right)$$

where $\theta_{B,R}$ is a known Bragg angle for a reference wavelength $\lambda_{R}$.

**Figure 4:**
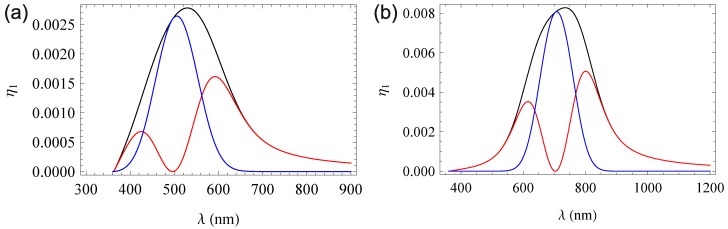
Diffraction efficiency for a Gaussian absorption band (**a**) and a polaronic absorption band (**b**). Each figure shows the overall diffraction efficiency (black) and the efficiency from the pure absorption (blue) and the pure refractive index grating (red).

Using the exemplary permittivities derived above, the curves shown in Figure 4 emerge.

Obviously, the induced refractive index change has a significant influence on the diffraction efficiency aside from the absorption maximum, while the contribution of both gratings is in the same order of magnitude in the ranges of their maximal amplitudes. The overall curve is a simple sum of the two pure gratings. Numerical analysis shows that the FWHM of the rocking curve is maintained over the complete spectrum under consideration. This is in strong agreement with the explanation for the lack of definition of the grating given in Section 3.1, as the effects involved are not wavelength dependent.

Notice that these effects are only necessarily true for phase-symmetrical gratings. We will examine non-phase-symmetric gratings in the following sections.

### 4.2. Effects of Phase Shifts in Mixed Gratings

For a pure refractive or absorption grating, the analysis of the grating parameters is trivial. The modulation depth of the particular grating can be derived from the in-Bragg diffraction efficiency $\eta_{1}=I_{s}\left(z\right)/I_{0}$ from Equations (36) and (37).

Only in the case that the effective grating thickness $d_{h}$ is smaller than the sample thickness *d*—for example through holographic recording in strongly absorbing materials—the rocking curve is required to determine the actual grating parameters in the form of an effective grating thickness, as the in-Bragg results only allow to determine the $z\epsilon_{1}^{\prime }\left(\lambda \right)$ product. However, they allow a decent estimation as a starting value for a fit using Equations (33) and (34).

**Figure 5:**
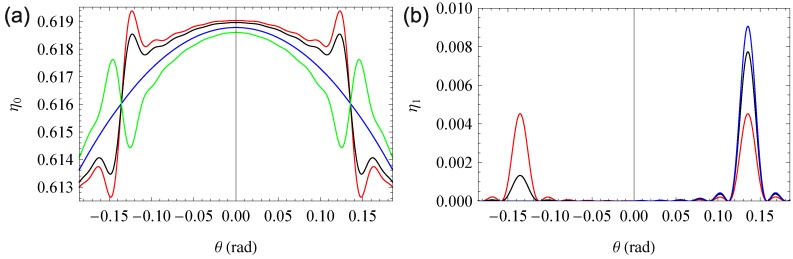
Zeroth (**a**) and first order (**b**) diffraction efficiency for $\epsilon_{1}^{\prime \prime }\left(\lambda \right)\;=\;\epsilon_{1}^{\prime }\left(\lambda \right)\;=\;1,13\times 10^{-3}\;{\mathrm{F\cdot m}}^{-1}$. Figure (**a**): Φ=0 (red), Φ=π/4 (black), Φ=π/2 (blue), Φ=3π/4 (green). Figure (**b**): Φ=0 (red), Φ=π/4 and Φ=3π/4 (black), Φ=π/2 (blue).

For mixed and potentially out-of-phase gratings, for which experimental evidence has been provided in [41], the situation is more complicated [42,43]. First theoretical results for a related Borrmann effect have been presented in [44] and a comprehensive discussion is given in [20] including a qualitative discussion of the Borrmann effect in the diffraction efficiency of the positive and negative range of the angle of incidence. Basically, out-of-phase gratings result in different first order diffraction efficiencies for positive and negative Bragg angles $\eta_{1}\left(\pm \theta \right)$ and a shift of the maximal zeroth order diffraction efficiency away from the Bragg angle. Examplary plots are given in Figure 5. We have chosen $\epsilon_{1}^{\prime \prime }\left(\lambda \right)=\epsilon_{1}^{\prime }\left(\lambda \right)$, as this configuration gives the most pronounced effects.

While the effect itself has been described qualitatively in the literature cited above, a quantitative theoretical description has not been presented yet. We will analyse the ratio of first order diffraction efficiencies between the positive and negative angle range in the following section.

Note that against this background, a quantity like the “Japanese diffraction efficiency” $\eta_{1}/\left(\eta_{0}+\eta_{1}\right)$ is meaningless as an intrinsic scalar attribute of the grating, as in such gratings, the value may strongly depend on the direction of incidence.

## 5. Quantitative Analysis of the Borrmann Effect

Due to the simplification of the formal representation presented above, the quotient $\eta_{1}\left(\theta_{B}\right)/\eta_{1}\left(-\theta_{B}\right)$ can be calculated very easily from Equation (35). As only the denominator in Equation (35) depends on the direction of the incident beam, we simply get

(43) $$\begin{array}{cc} \frac{\eta_{1}\left(\theta \right)}{\eta_{1}\left(-\theta \right)} & =\frac{\epsilon_{1}^{\prime \prime }{\left(\lambda \right)}^{2}+2\sin\left(\Phi \right)\epsilon_{1}^{\prime \prime }\left(\lambda \right)\epsilon_{1}^{\prime }\left(\lambda \right)+\epsilon_{1}^{\prime }{\left(\lambda \right)}^{2}}{\epsilon_{1}^{\prime \prime }{\left(\lambda \right)}^{2}-2\sin\left(\Phi \right)\epsilon_{1}^{\prime \prime }\left(\lambda \right)\epsilon_{1}^{\prime }\left(\lambda \right)+\epsilon_{1}^{\prime }{\left(\lambda \right)}^{2}}\\ & =\frac{1+Q_{\text{BA}}^{2}+2Q_{\text{BA}}\sin\left(\Phi \right)}{1+Q_{\text{BA}}^{2}-2Q_{\text{BA}}\sin\left(\Phi \right)}\\ & ={\left|\frac{\epsilon_{1}^{\prime }\left(\lambda \right)+ie^{-i\Phi }\epsilon_{1}^{\prime \prime }\left(\lambda \right)}{\epsilon_{1}^{\prime }\left(\lambda \right)+ie^{i\Phi }\epsilon_{1}^{\prime \prime }\left(\lambda \right)}\right|}^{2}\\ & ={\left|\frac{Q_{\text{BA}}+ie^{-i\Phi }}{Q_{\text{BA}}+ie^{i\Phi }}\right|}^{2} \end{array}$$

where $Q_{\text{BA}}=\epsilon_{1}^{\prime }\left(\lambda \right)/\epsilon_{1}^{\prime \prime }\left(\lambda \right)$. The same can be achieved using the complete off-Bragg Equation (32) resulting in the last two lines of Equation (43). Thus, the ratio is constant over the full rocking curve.

**Figure 6:**
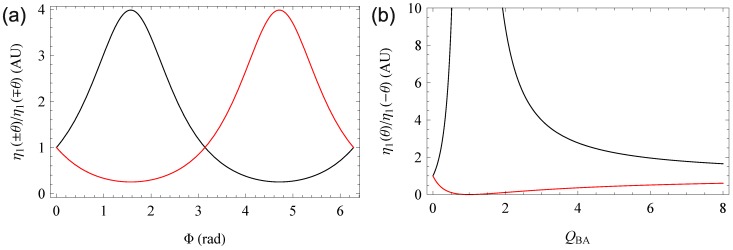
Proportions $\eta_{1}\left(\theta \right)/\eta_{1}\left(-\theta \right)$ (black) and $\eta_{1}\left(-\theta \right)/\eta_{1}\left(\theta \right)$ (red). In Figure (**a**), the phase shift Φ-depending ratios are show for $Q_{BA}=3$; In Figure (**b**) the $Q_{BA}$-depending ratios are given for Φ=π/2.

Equation (43) fulfils the necessary symmetry condition $f\left(\Phi \right)=f{\left(2\pi -\Phi \right)}^{-1}$ and reaches its maximum at Φ=π/2. This is expected as, in this case, the interference pattern of the reference and signal beam appears in its optimal position (*i.e*., in the position that would emerge in pure gratings) relative to both gratings in either the positive or the negative angular range. The corresponding graph is shown in Figure 6. Figure 6b shows a high signal-to-noise ratio for $Q_{BA}$ in the range of at least 0 to 5. For larger ratios between real and imaginary part of the permittivity, accurate measurements may be difficult. The pole at Φ=π/2 corresponds to the blue curve in Figure 5a, where the signal beam vanishes in the negative Bragg angle.

For a mixed grating ($\epsilon_{1}^{\prime \prime }\left(\lambda \right)\neq 0\neq \epsilon_{1}^{\prime }\left(\lambda \right)$), due to the symmetry of the sine, Equation (43) will always have a solution of the form $\Phi =\pi /2\pm \Delta \Phi (Q_{BA})$ or $\Phi =-\pi /2\pm \Delta \Phi (Q_{BA})$. This corresponds to the black curve in Figure 5a representing Φ=π/4 and Φ=3π/4. These two results may only be distinguished by regarding the zeroth order diffraction efficiency. It has been shown in [20] that |Φ|<π/2 if and only if $\eta_{0}\left(\theta =0\right)<A_{LB}\left(0\right)$.

**Figure 7:**
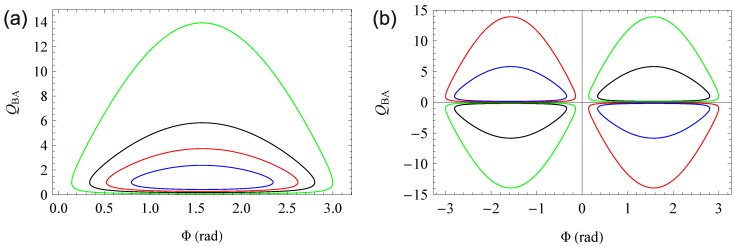
Graph of Equation (44). Figure (**a**) shows the relation between $Q_{\text{BA}}$ and Φ, for $\eta_{1}\left(\theta \right)/\eta_{1}\left(-\theta \right)=4/3$ (green), 2 (black), 3 (red), 6 (blue); Figure (**b**) shows the complete mathematical solution including $\eta_{1}\left(\theta \right)/\eta_{1}\left(-\theta \right)<1$ , for $\eta_{1}\left(\theta \right)/\eta_{1}\left(-\theta \right)=4/3$ (green), 2 (black), 3/4 (red), 1/2 (blue).

Equation (43) can be solved for $Q_{BA}$, resulting in

(44) $$Q_{\text{BA}}=\frac{\left(\frac{\eta_{1}\left(\theta \right)}{\eta_{1}\left(-\theta \right)}+1\right)\sin\left(\Phi \right)\pm \sqrt{{\left(\frac{\eta_{1}\left(\theta \right)}{\eta_{1}\left(-\theta \right)}+1\right)}^{2}\sin{\left(\Phi \right)}^{2}-{\left(\frac{\eta_{1}\left(\theta \right)}{\eta_{1}\left(-\theta \right)}-1\right)}^{2}}}{\frac{\eta_{1}\left(\theta \right)}{\eta_{1}\left(-\theta \right)}-1}$$

or for Φ, yielding

(45) $$\begin{array}{cc} \Phi & =\pi -\arcsin\left(\frac{\left(1+Q_{\text{BA}}^{2}\right)-\left(\frac{\eta_{1}\left(\theta \right)}{\eta_{1}\left(-\theta \right)}-1\right)}{2Q_{\text{BA}}-\left(\frac{\eta_{1}\left(\theta \right)}{\eta_{1}\left(-\theta \right)}+1\right)}\right)+2\pi N\;\text{or}\\ \Phi & =\arcsin\left(\frac{\left(1+Q_{\text{BA}}^{2}\right)-\left(\frac{\eta_{1}\left(\theta \right)}{\eta_{1}\left(-\theta \right)}-1\right)}{2Q_{\text{BA}}-\left(\frac{\eta_{1}\left(\theta \right)}{\eta_{1}\left(-\theta \right)}+1\right)}\right)+2\pi N \end{array}$$

where N is an integer. The two solutions for Φ may be distinguished as mentioned above. Obviously, only positive quotients $Q_{\text{BA}}$ have physical nature, resulting in Figure 7. For $\eta_{1}\left(\theta \right)/\eta_{1}\left(-\theta \right)\to 1$, the loop tends towards a rectangular curve delimited be the axes and Φ=π and $Q_{\text{BA}}=\infty$. This proves that a first order diffraction efficiency symmetric with respect to the read-out angle is only achievable with pure gratings or symmetric gratings (*i.e*., Φ=0 of Φ=π).

To determine all three independent grating parameters $\epsilon_{1}^{\prime }$, $\epsilon_{1}^{\prime \prime }$ and Φ, at least three independent data points are needed. In-Bragg, these could be $\eta_{1}\left(\pm \theta_{B}\right)$ and $\eta_{0}\left(\theta_{B}\right)$. Unfortunately, as shown in Figure 5 (where $\eta_{0}\left(\theta_{B}\right)$ is nearly the same for all Φ), $\eta_{0}\left(\theta_{B}\right)$ has a very low signal-to-noise ratio and thus does not qualify for the examination. Consequently, either off-Bragg or a different setup, for example involving two incident beams, is necessary.

Finding an analytical expression for the shift of the extrema around the Bragg angle of Equation (21) is incomparably harder than the previous calculations. The equation has the structure

(46) $$\eta_{0}=\sin{\left(k(\theta )z\right)}^{2}+\left(1+\Delta (\theta )\right)\cos{\left(\left(k(\theta )+\Delta k(\theta )\right)z\right)}^{2}$$

where Δ(θ),Δk(θ)≪1 and thus $\eta_{0}\approx 1$ according to $\sin{\left(x\right)}^{2}+\cos{\left(x\right)}^{2}=1$. Thus, it is not enough to examine only a part of the full term.

Currently, the most efficient way for the examination of the grating appears to be inserting Equation (45) into Equations (35) and (39) at $\theta =\theta_{\text{max}}$—reducing the parameter space to two dimensions—and solving for $\epsilon_{1}^{\prime \prime }\left(\lambda \right)$, $\epsilon_{1}^{\prime }\left(\lambda \right)$ numerically.

## 6. Conclusions

Until recently, analyses in the field of holographic spectroscopy have not been necessary in electro-optics, as the fundamental mechanisms show little dispersion characteristics. However, methods like the absorption-induced change of the refractive index and new photosensitive materials have brought wavelength-dependent holograms with pronounced photosensitivities over the entire optical spectrum range into the focus of attention.

Kogelnik’s coupled-wave theory is still a valuable basis for the study of these topical problems. However, the original works make assumptions that do not comply with all modern experimental setups. Successive works closed some of these gaps at the cost of significantly increased algebraic complexity. The set of equations presented here reduces this complexity to a level that allows advanced analysis of even those effects that occur only in complex gratings (Borrmann effect). As an example, the analytic tools supplied in this paper are applied not only to describe the dispersion behaviour of such gratings, but also to investigate dependencies on the direction of incidence on out-of-phase mixed gratings, that can be observed, e.g., in holographic polymer dispersed liquid crystals. Using our quantitative analysis, it is possible to determine the full set of grating parameters (real and imaginary part of the permittivity modulation, and phase shift between these modulations) of such systems.

## Nomenclature of Material Properties

*ϵ*(**x**, *λ*): Complex permittivity
$\epsilon_{0}^{\prime }\left(\lambda \right)$: Mean real permittivity
$\epsilon_{0}^{\prime \prime }\left(\lambda \right)$: Mean imaginary permittivity
$\epsilon_{1}^{\prime }\left(\lambda \right)$: Amplitude of real permittivity grating
$\epsilon_{1}^{\prime \prime }\left(\lambda \right)$: Amplitude of imaginary permittivity grating
Φ_*B*_: Phase offset of real permittivity grating compared with interference pattern at z = 0
Φ_*A*_: Phase offset of imaginary permittivity grating compared with interference pattern at z = 0
Φ: Φ_*A*_ − Φ_*B*_ (for diffraction experiments with no interference pattern at z = 0)
**n**(**x**, *λ*): Complex refractive index
*α*_0_(*λ*): Mean absorption coefficient
*n*(**x**, *λ*): Real refractive index
*n*_0_(*λ*): Mean real refractive index
*n*_1_(*λ*): Amplitude of real refractive index grating
Φ_*n*_: Phase offset of real refractive index grating
*κ*_0_(*λ*): Mean absorption index
*κ*_1_(*λ*): Amplitude of grating
Φ_*κ*_: Phase offset of absorption index grating
**K**: Grating vector in material

## Nomenclature of LightWave Properties

*R*(*z*)/*S*(*z*): Electric field amplitude of reference/signal beam inside material
*R*_0_/*S*_0_: Intial electric field amplitude of reference/signal beam inside material
**e**_*R*_(*z*)/**e**_*S*_(*z*): Polarisation unit vector of reference/signal beam
Φ_Diff_: Phase difference between reference and signal beam inside the material
**k**: Wave vector of reference beam inside material
Φ_0_: Phase shift of interference pattern between reference/signal beam
*m*: Modulation depth of interference pattern
*I/I_R_/I_S_*: Intensity of interference pattern/reference beam/signal beam
*η*_0_/*η*_1_: Transmission efficiency/diffraction efficiency
